# 

**Published:** 1849-05

**Authors:** 


### THE WESTERN JOURNAL OF MEDICINE AND SURGERY:

EDITED BY

LUNSFORD P. YANDELL, M. D.,

PROFESSOR OF CHEMISTRY AND PHARMACY IN THE UNIVERSITY OF LOUISVILLE.

AND

THEODORE S. BELL, M. D.

NO. CXIII.—MAY, 1849.

**THIRD SERIES.**

Vol. III.....No. V.

LOUISVILLE, KY:

PUBLISHED MONTHLY BY PRENTICE AND WEISSINGER,

PROPRIETORS AND PRINTERS.

1849.

[POSTAGE 6¼ CENTS.]

